# The Second Law of Infodynamics: A Thermocontextual Reformulation

**DOI:** 10.3390/e27010022

**Published:** 2024-12-30

**Authors:** Harrison Crecraft

**Affiliations:** Independent Researcher, Leesburg, VA 20176, USA; harrison@crecraft.net

**Keywords:** entropy, origin of life, irreversible thermodynamics, MaxEnt, general evolution, information, statistical mechanics

## Abstract

Vopson and Lepadatu recently proposed the Second Law of Infodynamics. The law states that while the total entropy increases, information entropy declines over time. They state that the law has applications over a wide range of disciplines, but they leave many key questions unanswered. This article analyzes and reformulates the law based on thermocontextual interpretation (TCI). The TCI generalizes Hamiltonian mechanics by defining states and transitions thermocontextually with respect to an ambient-temperature reference state. The TCI partitions energy into exergy, which can do work on the ambient surroundings, and entropic energy with zero work potential. The TCI is further generalized here to account for a reference observer’s actual knowledge. This enables partitioning exergy into accessible exergy, which is known and accessible for use, and configurational energy, which is knowable but unknown and inaccessible. The TCI is firmly based on empirically validated postulates. The Second Law of thermodynamics and its information-based analog, MaxEnt, are logically derived corollaries. Another corollary is a reformulated Second Law of Infodynamics. It states that an external agent seeks to increase its access to exergy by narrowing its information gap with a potential exergy source. The principle is key to the origin of self-replicating chemicals and life.

## 1. Introduction

Melvin Vopson and S. Lepadatu introduced the Second Law of Infodynamics in an article they published in 2022 [1]. They resolve a system’s total entropy into physical entropy and information entropy. The total entropy of an isolated system (or system plus surroundings) is constant or increases over time, in compliance with the Second Law of thermodynamics. According to the Second Law of Infodynamics, however, a system’s information entropy remains constant or declines over time. Vopson and Lepadatu state that the Second Law of Infodynamics has important applications in diverse fields, including genetics, evolutionary biology, virology, computing, big data, physics, and cosmology, but they acknowledge important unresolved questions. The aim of this article is to resolve their unanswered question and to put information and physical entropies and a reformulated law of infodynamics on a firm conceptual foundation.

A system’s total entropy can be statistically defined by $\sigma =-\sum_{i=1}^{N}P_{i}ln(P_{i})$, where P_i_ is the probability of the system being in the mechanical microstate i, and N is the number of microstates available to the system. Vopson and Lepadatu resolve total entropy into physical entropy and information entropy. Physical entropy corresponds to Gibbs entropy of thermodynamics. It is summed over thermalized microstate potentialities, which are random and therefore have no information content. Information entropy, in contrast, is summed over information-bearing microstates. Information-bearing microstates are not random; they are definite but unknown. They reveal their information content by measurement and observation. Information entropy is the entropy of statistical mechanics.

Information entropy is closely related to Shannon entropy, as defined by Claude Shannon in his seminal article on information theory [2]. Shannon entropy describes the average level of surprise or uncertainty inherent to a variable’s possible outcomes upon its measurement and observation [3]. The surprise factor is a measure of the information gap between a system’s actual information content and an observer’s expectations.

Vopson and Lepadatu provide two examples to illustrate the spontaneous decline in information entropy. The first example describes the decay of a magnetically stored digital record with definite, but unknown, content. Magnetic relaxation reduces the storage medium’s information content, and over time, this reduces an observer’s surprise factor upon reading it.

The second example describes the change in the information entropy of SARS-CoV-2 recorded in its RNA between January 2020 and October 2021. The virus’s RNA comprises four bases (adenine, cytosine, guanine, and uracil), with a total connected length of n = 29,903 bases. Entropy is summed over the $N=\frac{n!}{n_{A}!n_{C}!n_{G}!n_{U}!}$ different linear configurations of the four bases. Based on equal numbers of each base and Sterling’s approximation, the maximum theoretical information entropy is σ = −n*log_2_*(¼) = 59,806 bits. Vopson and Lepadatu’s Figure 3 [1] shows a decline in the information entropy of the RNA over the period from 40,568 to 40,553 bits.

The decline in information entropy in the first example is a direct consequence of the thermalization and randomization of the magnetic moments of the storage medium. Thermal equilibration is based on the Second Law of thermodynamics. However, the Hamiltonian conceptual framework (HCF), on which classical and quantum mechanics are interpreted, does not formally recognize entropy or irreversibility as fundamental properties. It cannot formally recognize either the Second Law of thermodynamics or the Second Law of Infodynamics as fundamental laws of physics.

The decline in information entropy in the SARS virus example is not immediately expected. Unbiased random mutations by themselves would be expected to increase a system’s information entropy. This raises a deeper question about the definition and meaning of information entropy. E. T. Jaynes [4] emphasized the incompatibility between randomness and mechanics. He concluded that information entropy is based on an observer’s incomplete knowledge. He further concluded that the observer’s assignment of probabilities represented their subjective expectations and bias. To eliminate subjective observer bias, Jaynes asserted that initial probabilities should be based on zero prior information. With zero information, all configurations have equal-probability expectations. This is the statistical mechanical interpretation of entropy, and it is the information entropy of Vopson and Lepadatu.

One alternative to Jaynes’s procedure for assigning initial probabilities is to base expectations on a system’s most recent measurement. If zero measurement error and no mutations are assumed, then a system’s entropy following measurement and observation would be zero. This represents a perfectly precise description, but it almost certainly would not be accurate. A more realistic statistical description would be to assign a positive entropy to reflect measurement error and random changes. Another alternative would be to assume thermal equilibrium and calculate the Gibbs entropy based on the configurations’ energies and the Boltzmann–Gibbs probability distribution function [5].

It is clear that statistical entropy is subjectively based on an observer’s prior knowledge or on arbitrary assumptions. It is ill defined, and it is not suitable to define a physical law. Nevertheless, the evolution of a virus does, in some sense, represent a gain in information, reflecting its evolving ability to evade antibodies and to manipulate its target genomes for its own reproduction. A well-defined principle describing nature’s tendency to acquire and act on information would be key to understanding the origin and evolution of life.

This paper reformulates the Second Law of Infodynamics within the framework of the thermocontextual interpretation (TCI). The TCI was previously introduced as an alternative to Hamiltonian mechanics [6]. The TCI defines the state of a system thermocontextually with respect to a reference state in equilibrium with the ambient surroundings at a positive ambient temperature. It recognizes entropy, exergy, and thermal energy (heat) as physical properties of states. The TCI provides a generalized framework for mechanics and thermodynamics, and it resolves some of the fundamental questions of physics regarding time, causality, and quantum entanglement [6].

The TCI, like thermodynamics, defines irreversible change with respect to a fixed reference by the dissipation of exergy or, equivalently, by the production of entropy. The irreversible approach toward equilibrium, however, is fundamentally deterministic. Determinism means that information is conserved, and this precludes addressing changes in information or the Second Law of Infodynamics. To address infodynamics, the TCI is updated here to accommodate spontaneous random changes in addition to irreversible changes.

The TCI is updated here to describe generalized transitions, which can result from changes in the system, changes in the system’s environment, or both. The updated TCI contextually defines transitions with respect to an observer’s fixed reference and measurement framework. These changes take the TCI well beyond classical thermodynamics, as detailed by Lieb and Yngvason [7] and by Gyftopoulos and Beretta [8]. Classical thermodynamics focuses on energy states and on the ordering of states by irreversible but deterministic transitions, as defined by increasing thermodynamic entropy or dissipation. The determinism of thermodynamics and mechanics precludes their formal accommodation of changes in information. The updated TCI provides a formal framework for describing configurational information and measurements. It accommodates the measurement of new information following its spontaneous creation by random instantiations of microstate potentialities to definite but unknown microstate actualities.

The TCI is firmly based on empirically validated postulates. The Second Law of thermodynamics, its information-based analog MaxEnt [4,9], and an objectively defined reformulation of the Second Law of Infodynamics are all logically derived corollaries of the TCI’s updated postulates.

## 2. The Two Entropies

Rudolf Clausius introduced entropy in 1850 and defined entropy change by the ratio of heat (q) and absolute temperature (T):

(1) $$dS\equiv \frac{dq}{T}.$$

Clausius then proceeded to formulate the Second Law of thermodynamics in terms of the irreversible increase in thermodynamic entropy. His formulation was in the spirit of Sadi Carnot’s conjecture based on his work on steam engines. Carnot concluded that the actual output of work is less than a system’s idealized potential for work due to irreversible losses. The Second Law states that while total energy is conserved, work potential is irreversibly dissipated to the heat of the ambient surroundings. It further states that ambient heat has zero potential for work on the ambient surroundings.

Shortly after Clausius defined thermodynamic entropy, Ludwig Boltzmann defined entropy statistically within the Hamiltonian framework of classical mechanics. He defined entropy by the number of mechanical microstate configurations consistent with a system’s thermodynamic macrostate description. In the idealized dissipation-free world of Hamiltonian mechanics, however, all information and work potential are conserved. There is no distinction between past and future, and there is no arrow of time. The HCF defines a system’s physical state by perfect measurement in the absence of thermal noise, and it provides the foundation for the current interpretations of classical, quantum, and relativistic mechanics.

Following Boltzmann’s initial formulation of statistical entropy, Willard Gibbs applied it to thermally equilibrated systems and defined what is now known as Gibbs entropy:

(2) $$S_{Gibbs}\equiv -k_{B}\sum_{i=1}^{N}P_{i}\ln\left(P_{i}\right),$$

where k_B_ is Boltzmann’s constant, N is the number of energy states, and P_i_ is the probability that a measurement reveals a microstate with energy E*_i_*. Gibbs entropy is equal to the Third-Law entropy of thermodynamics, where the Third-Law entropy is defined with respect to absolute zero:

(3) $$S_{Gibbs}=S_{3rdL}\equiv \int_{0K}^{T_{sys}}\frac{dq}{T}=\int_{0K}^{T_{sys}}\frac{C_{v}dT}{T}.$$

where $C_{v}={\left(\frac{\partial q}{\partial T}\right)}_{v}$ is the volumetric heat capacity and a property of state. The Gibbs entropy and Third-Law entropy are expressed in units of energy per kelvin, and they are denoted by the letter “S”.

The Third-Law entropy (3) is a thermodynamic property of a thermally equilibrated state. Gibbs entropy, in contrast, expresses the statistical distribution of energy measurements. The empirical equality of Gibbs and Third-Law entropies is the basis for the Boltzmann–Gibbs distribution function [5]. The Boltzmann–Gibbs distribution defines the probability P_i_ of measuring a microstate with energy E*_i_* by the following:

(4) $$P_{i}=e^{\frac{{-E}_{i}}{k_{B}T}}/Z\;with\sum_{i=1}^{N}P_{i}=1.$$

where T is the equilibrium temperature of thermalization, and Z is a normalization factor for probabilities to sum to one. The higher a microstate’s energy is, the lower is its probability of being measured.

It is universally accepted that the entropy of an isolated system (or system plus surroundings) spontaneously increases and never decreases. However, increasing entropy describes two fundamentally different processes: dissipation and dispersion. The Second Law of thermodynamics addresses the irreversible dissipation and production of thermodynamic entropy by dissipative processes such as heat flow or friction. The statistical mechanical analog of the Second Law, MaxEnt, in contrast, describes dispersion and the spontaneous increase in an observer’s uncertainty of a system’s microstate configuration. Classical mechanics attributes the statistics of dispersion to classical chaos and to an observer’s subjective uncertainty of a system’s initial microstate.

Classical mechanics is known to break down at low temperatures and small scales, and quantum mechanics defines the quantum microstate by a pure wavefunction. Extending the classical concept of a microstate as being completely defined by perfect measurement, quantum mechanics defines the quantum state tomographically as an ensemble of all the possible measurements [10]. The wavefunction defines a pure quantum state with zero uncertainty and zero statistical mechanical entropy.

Quantum mechanics describes wavefunction collapse as the transition from a pure superposed microstate configuration to a “mixed state”. A mixed state is defined by a statistical ensemble of measurable microstate possibilities following wavefunction collapse. The statistics of a mixed state’s measurement results are expressed by the von Neumann entropy. However, the intrinsic determinism of the Schrödinger wavefunction does not formally accommodate randomness or wavefunction collapse. Quantum mechanics does not address whether the statistics of quantum measurements reflect actual physical randomness, whether they reflect hidden variables, or whether they are triggered by observation. Interpretations of quantum randomness are actively debated [11] and there is no consensus interpretation.

Claude Shannon significantly extended the application of statistical entropy with his publication *A Mathematical Theory of Communication* in 1948 [2]. He introduced Shannon entropy to describe a message receiver’s uncertainty of a statement’s precise meaning. Shannon entropy is mathematically identical, up to a constant multiplier, to Gibbs entropy and to statistical mechanical entropies. All are specific cases of a generalized statistical entropy, which can be defined by the following:

(5) $$\sigma \equiv -\sum_{i=1}^{N}P_{i}\ln\left(P_{i}\right).$$

where N is the number of states, whether they be messages, energy states, or mechanical microstate configurations. P_i_ is the probability that a measurement will reveal microstate *i*. Statistical entropy is unitless, and we denote statistical entropy by sigma (σ). As defined, statistical entropy only describes the statistics and uncertainty of the observation results. It does not offer a physical interpretation of the probabilities.

We can also express thermodynamic entropy as a unitless statistical entropy, which we denote here as thermal entropy and define by the following:

(6) $$\sigma_{q}\equiv S_{Gibbs}/k_{B}.$$

Thermal entropy is based on Boltzmann–Gibbs probabilities (4), which describe the statistical distribution of energy observations for thermalized microstates.

Thermodynamics and statistical mechanics both describe spontaneous increases in entropy, but they differ fundamentally in their interpretation of entropy. Thermodynamics interprets entropy as the thermal randomness of thermalized energy states. Mechanics, in contrast, does not recognize objective randomness. It interprets the increase in informational entropy as an observer’s increased uncertainty of the actual microstate configuration, which is definite and evolves deterministically. E.T. Jaynes referred to the increase in information entropy as MaxEnt [4].

The Hamiltonian conceptual framework is the universally accepted framework for interpreting mechanics—classical, quantum, and relativistic. It regards thermodynamics as an incomplete description, and entropy and irreversibility as the subjective properties of perception, rather than as the objective properties of physical states. But at the same time, it recognizes the Second Law of thermodynamics and MaxEnt as two descriptions of a thoroughly validated empirical law. As pointed out by Takahiro Sagawa, however, there is no a priori relationship between thermodynamic entropy and informational entropy [12]. Thermodynamic and information entropies are two entirely different entropies, and their increases reflect two distinct empirically validated laws.

## 3. The Thermocontextual State

Crecraft introduced the thermocontextual interpretation (TCI) as an alternative to the Hamiltonian conceptual framework (HCF) in “Time and Causality: A Thermocontextual Perspective” [6]. The article defined system time as a complex property of state spanning irreversible thermodynamic time and reversible mechanical time. Mechanical time is as described in mechanics: a coordinate of a state’s position along a reversible trajectory. Thermodynamic time is measured by the dissipation of energy (or equivalently by the production of thermodynamic entropy). The TCI also recognizes reference time, a property of a system’s surroundings as measured by an external clock. Reference time is the time of relativity. It distinguishes the future light cone from the past light cone, and it defines the arrow of relativistic causality. The article provided a simple explanation for Bell-type experiments [13], which document the nonlocal superluminal correlations of measurements on spatially separated entangled particles. The TCI provides a commonsense explanation of Bell-type experiments without the need for splitting universes, hidden variables, superluminal action, or superdeterminism, which are typically invoked to explain the experimental results.

### 3.1. TCI Postulates of State

TCI’s description of states is based on the laws of physics, which includes thermodynamics’ First Law of energy conservation, and the following additional postulates:

**Postulate 1:**  *(0th Law of thermodynamics). Temperature is a measurable property of state.*

**Postulate 2:**  *(Based on the Third Law of thermodynamics). A system’s surroundings are always at a positive temperature.*

**Postulate 3:**  *No hidden variables—a system’s state properties are observable by perfect measurement and observation.*

Postulates One and Two are directly based on the Zeroth and Third Laws of thermodynamics. The Zeroth Law was the last thermodynamic law formalized in order to establish temperature as a measurable property. The Third Law states that absolute zero temperature can never be reached. Postulates One and Two establish that the minimum temperature of a system’s surroundings is always positive. The TCI defines the minimum temperature of the surroundings that the system can potentially interact with as the system’s positive ambient temperature and a measurable property of state.

Postulate Three states that there are no hidden variables. Postulate Three is an explicit rejection of an assumption for which there can be no empirical justification. It means that there exists, in principle, a perfect observer that can measure a system’s complete state with zero uncertainty.

It is important to note, however, that Postulate Three does not imply that a system always exists as a state. The logically equivalent contrapositive of Postulate Three is that if a system’s state is not completely observable, then there is no state. This describes a system in transition between states. A state can exist as a kinetically frozen metastable system or as a system in complete equilibrium with its fixed ambient surroundings. A non-equilibrium state can spontaneously transition to a more stable microstate, during which it is in transition between states, and it does not exist as a state.

With no hidden variables, the statistical distribution of quantum measurements cannot be deterministically tied to unobservable state properties. Consequently, measurements are not just statistical, they are intrinsically random. Postulate Three therefore establishes the physical randomness of transitions, as expressed by Corollary 3-1:

**Corollary 3-1.**  *The statistics of measurements reflect the intrinsic randomness of the measurements.*

We note that randomness does not apply to the physical microstate, which is observable and therefore always definite. It applies to a microstate’s potentialities, which are virtual microstates that can be randomly instantiated during transition to a new definite state, such as the instantiation of an observable measurement result.

Postulates One to Three provide a unified framework for defining a system’s thermocontextual state, as described in the following sections. The postulates apply equally well to classical, quantum, and thermodynamic states.

### 3.2. Thermocontextual Properties of State

Thermocontextual properties of state are defined relative to a system’s ambient reference state (ARS) [6,14]. The ARS is defined by the system’s equilibrium state at the ambient temperature and pressure of the surroundings. The ambient temperature and pressure are defined by the minimum temperature and pressure of the surroundings, with which the system can interact. The ambient surroundings are defined as the idealized equilibrium surroundings at the ambient temperature and pressure.

State properties are defined with respect to the ARS, but they are empirically determined by measurements. We, therefore, also need to address the reference for measurements. When a technician inserts a thermometer into an ice bath to measure its temperature, the thermometer is part of the external measurement setup and physical surroundings. The probe’s temperature defines the observer’s reference temperature for measuring the system’s temperature. Since the ambient temperature is defined as the minimum external temperature with which the system can interact, the ambient temperature cannot exceed the external probe’s reference temperature (T_a_ ≤ T_ref_). Similarly, the probe’s temperature cannot exceed the system temperature. (T_ref_ ≤ T_sys_). Otherwise, the probe’s greater thermal randomness would preclude perfect measurement and observation, which is codified by Postulate Three. This leads to the following relationships:

(7) $$T_{a}\leq T_{ref}\leq T_{sys}.$$

Likewise, for pressure, we have the following:

(8) $$P_{a}\leq P_{ref}\leq P_{sys}.$$

For perfect measurement of state properties, the reference and ambient surroundings are in equilibrium, and they have equal temperature and pressure.

The TCI resolves a system’s total energy, E_tot_, into system energy and ambient-state energy:

(9) $$E_{tot}=E+E_{as}\geq E_{as}\equiv \int_{0}^{T_{a}}C_{v}dT>0.$$

System energy, E, is defined relative to the ARS, with ambient-state energy, E_as_, defined with respect to absolute zero temperature. The ambient-state energy is the minimum-energy state for the system; there is no lower temperature or energy for the system to interact with. The ambient temperature and ambient-state energy are the objective thermocontextual properties of state, and they are always positive.

The TCI partitions system energy E into exergy X and entropic energy Q:

(10) E=X+Q.

**Figure 1:**
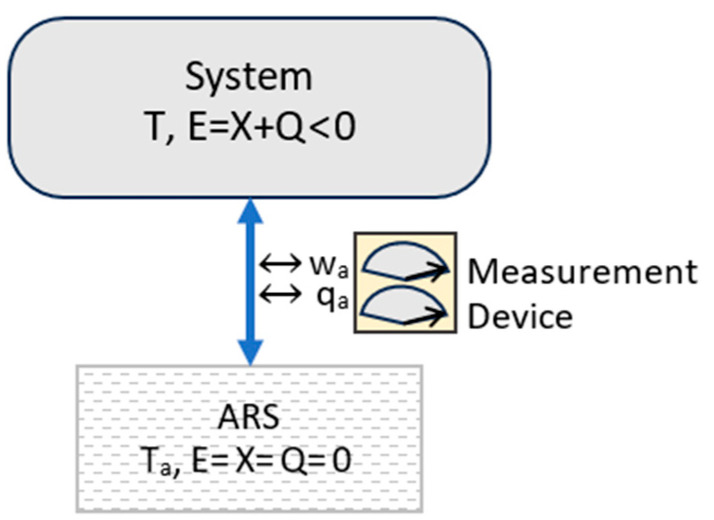
Perfect measurement of exergy and entropic energy. Perfect measurement is defined with respect to a measurement device in equilibrium with the ambient surroundings. As the system reversibly transitions to its equilibrium ambient state, exergy and entropic energy are the outputs and are recorded by a classical measurement device as exchanges of ambient work w_a_ and ambient heat q_a_. Work can be measured by the reversible lifting of a weight in a gravitational field. Absorbed heat can be measured by the work of expansion as a gas isothermally absorbs heat and maintains a fixed ambient temperature and energy.

Exergy and entropic energy are defined by perfect measurements of ambient work and ambient heat as a system reversibly transitions to its ARS (Figure 1). Ambient work is defined as the sum of mechanical work and work potential output to the ambient surroundings. Exergy equals the ambient work and entropic energy equals ambient heat, as reversibly recorded by an external measurement device. The measurement device is a macroscopic classical device in equilibrium with the ambient surroundings, and its record of results is independent of observation or any specific observer.

System energy can include external exergy, which is defined here as the sum of kinetic and potential energies with respect to an external reference frame. A system’s internal energy, U, is defined by subtracting external exergy from its system energy:

(11) $$U\equiv E-X_{ext}.$$

System energy can also include thermal energy (heat). Thermal energy is defined with respect to the ARS, and is given by the following:

(12) $$q=\int_{T_{a}}^{T}C_{v}dT.$$

For a non-isothermal system, thermal energy is defined by summing or integrating Equation (12) over subsystems at different temperatures. A system’s internal energy is increased by the addition of ambient work and heat:

(13) $$dU=dw_{a}+dq_{a}.$$

where dw_a_ and dq_a_ are increments of work and heat that are added to the system from the ambient surroundings.

Just as Equation (10) parses the system energy into exergy and entropic energy, we can parse internal energy into internal exergy and entropic energy:

(14) $$U=X_{U}+Q,$$

where internal exergy X_u_ equals X−X_ext_. We can then resolve internal exergy into mechanical exergy and thermal exergy:

(15) $$X_{U}=X_{m}+X_{q}.$$

Thermal exergy X_q_ is the idealized work potential of heat, and mechanical exergy X_m_ is the system’s work potential after thermally equilibrating with the ambient surroundings, at which point the system energy has no thermal energy. Mechanical exergy, such as the potential energy of a raised weight, is one hundred percent accessible for work. Mechanical energy is the energy of mechanics, and it is quantified by, and reversibly interchangeable with, mechanical work.

From elementary thermodynamics of the Carnot cycle and (12), thermal exergy is given by the following:

(16) $$dX_{q}=\frac{\left(T-T_{a}\right)}{T}dq=\frac{\left(T-T_{a}\right)}{T}C_{v}dT,$$

where dq is an increment of added thermal energy, T is its temperature of thermalization, and T_a_ is the fixed ambient temperature at which work is measured.

Combining (14) to (16), we obtain the following:

(17) $$dU=dX_{m}+dQ+dq-\frac{T_{a}}{T}dq,$$

and subtracting (13) from (17), we obtain the following:

(18) $$dQ=\frac{T_{a}}{T}dq,$$

where we used the equality between work and mechanical exergy. The TCI then defines entropy with respect to the ARS at the ambient temperature by the following:

(19) $$S_{TC}\equiv \int_{T_{a}}^{T}\frac{dq}{T}=\int_{T_{a}}^{T}\frac{dQ}{T_{a}}=\frac{Q}{T_{a}}.$$

Thermocontextual entropy is a generalization of the Third-Law entropy, which is defined relative to a hypothetical “ambient” reference at absolute zero temperature (3). The equalities in (19) are based on (18), and they relate entropic energy to entropy. It immediately follows from (19) and (3) that thermocontextual entropy and thermodynamic entropies differ only by their zero points, and that changes in the entropies are identical:

(20) $$\Delta S_{TC}=\Delta S_{Gibbs}=\Delta S_{3rdL}.$$

From (14) and (19), we obtain the following:

(21) $$U=X_{u}+Q=X_{u}+T_{a}S,$$

where we drop the TC subscript for entropy.

Equation (21) is the thermocontextual generalization of the thermodynamic relationship:

(22) U=F+TS,

where U is internal energy and F is Helmholtz free energy. Exergy and free energy are closely related, but whereas TS and free energy are defined only for isothermal systems at the system temperature T, exergy and entropic energy are defined with respect to a fixed ambient temperature, T_a_, and they are easily definable for non-isothermal systems.

Thermal energy, exergy, entropic energy, and entropy are all thermocontextual properties of state, defined with respect to the fixed ambient reference state, which specifies the ambient temperature and zero values for energy and entropy. They are well defined, and unlike thermodynamic properties, they are readily applicable to non-isothermal and far-from-equilibrium systems. The thermocontextual properties of state are summarized in Appendix A. Going forward, we assume that the system as a whole is at rest with its reference frame and external fields, so we can ignore external energy.

### 3.3. Energy States

A system’s energy state specifies the system’s exergy and entropic energy with respect to the ARS in equilibrium with the ambient surroundings. There are two requirements for an energy state:

1. The system is isolated from exchanges with the surroundings. Isolation means that it is fixed in energy and composition.
2. The system is non-dissipative. This means that there is no irreversible dissipation of exergy to ambient heat.

Requirements 1 and 2 fix the energy state’s composition, exergy, and entropic energy, but they do not fix the physical system’s microstate configuration. A system can reversibly transition across multiple degenerate microstate configurations sharing the same energy state.

**Figure 2:**
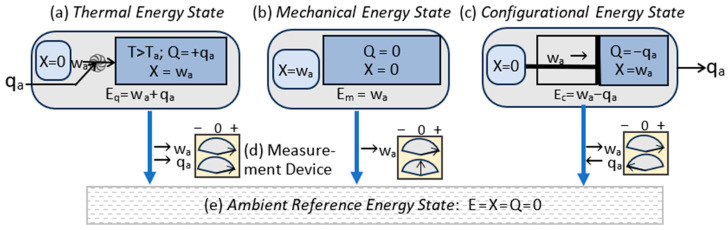
TCI energy states. Each system is prepared by applying work w_a_ to an ambient gas with zero stored exergy and E = X = Q = 0. (a) The thermal energy state is prepared by the work of reversibly pumping heat from the ambient surroundings into the gas. (b) The mechanical energy state is prepared by applying work to the mechanical battery only. (c) The configurational energy state is prepared by the work of isothermally compressing the gas. (d) Measurement devices record the exergy and entropic energy changes during reversible transitions back to the ARS. (e) The ARS defines the zero-energy levels for exergy and entropic energy. All the transitions are with respect to fixed ambient temperature and pressure.

Figure 2 illustrates three classifications of energy states based on whether their entropic energy content is positive, zero, or negative. Each energy state contains an ambient-gas cylinder and a mechanical “battery”, such as a spring or weight, to store mechanical exergy. Each state is prepared with ambient work w_a_ equal to nk_B_T_a_*ln*(2), which is the work needed to reversibly compress an ideal n-particle ambient gas to one-half of its initial volume.

The thermal energy state (a) is created by the work of reversibly pumping ambient heat up-temperature into the gas cylinder, increasing its temperature and entropic energy. The applied work equals the gas’s thermal exergy, and the pumped ambient heat equals its positive entropic energy. The thermal exergy can be accessed for work by rejecting the entropic energy back to the ambient surroundings via a reversible heat engine.

The mechanical energy state (b) stores its energy as mechanical exergy in its battery with zero entropic energy and zero randomness. The mechanical state energy has no entropic energy, and it is immediately accessible for external work independent of the ambient temperature.

The configurational energy state (c) is reversibly created by the work of isothermally compressing the gas to half volume. The gas’s temperature does not change during isothermal compression, and the gas’s energy is, therefore, fixed. The gas’s gain in exergy from the work of compression is offset by an equal rejection of entropic energy to ambient heat. The exergy can be accessed for work by reversibly running the piston in reverse.

Each system’s energy state in Figure 2 is defined by the exchanges of ambient work and ambient heat with an ambient measurement device as the system reversibly transitions back to the ARS. The three energy states in Figure 2 have the same exergy, equal to the applied work. They differ in whether their energy is mechanical-state energy **E_m_**, with Q = 0; thermal-state energy **E_q_**, with Q > 0; or configurational-state energy **E_c_**, with Q < 0.

**Figure 3:**
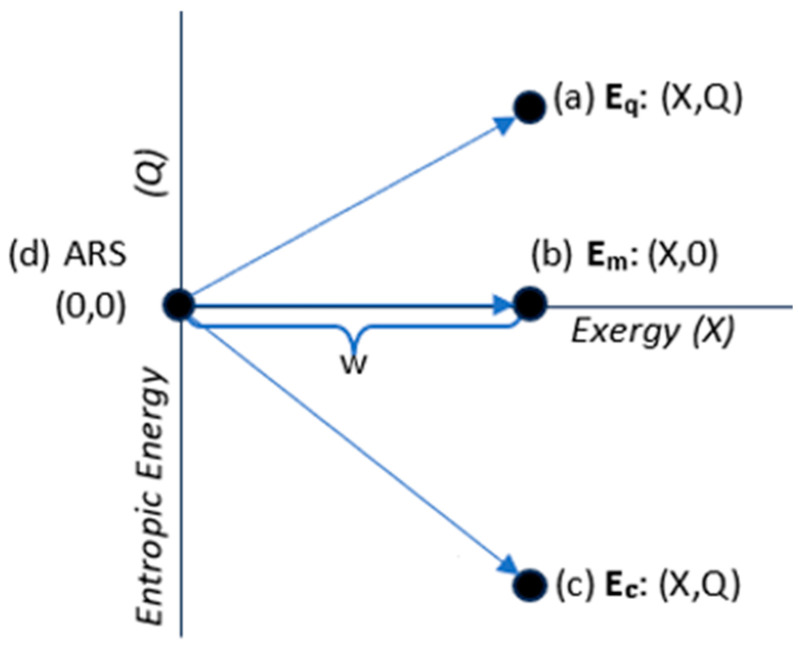
TCI energy states and their preparations in an X−Q space. (a) The thermal state’s energy, **E_q_**, has an exergy equal to its work of preparation, w, and positive entropic energy, Q, equal to the ambient heat reversibly pumped from the ambient surroundings. (b) The mechanical state’s energy, **E_m_**, has an exergy equal to its work of preparation and zero entropic energy. (c) The configurational state’s energy, **E**_c_, has an exergy equal to its applied work, w, and negative entropic energy, equal to the ambient heat expelled during isothermal compression. (d) Measurements of the energy states are defined by the changes in measurable work and ambient heat as the systems reversibly transition to the ARS.

The energy states in Figure 2 are plotted in Figure 3 in an X–Q space. The arrows in Figure 3 show the preparation of the states, starting from the zero-energy ambient state and applying work and adding or removing ambient heat. Measurement is simply the reverse transition to the ambient reference state, with a reversible record of the changes.

Figure 3 highlights the fact that an energy state’s energy is not a simple scalar. It is two-dimensional, spanning exergy and entropic energy. We denote 2D energy by boldfaced **E**:

(23) $$E=\left(X,Q\right).$$

where the magnitude of 2D energy is equal to the simple sum of its scalar components, E = X + Q, as was given in (10). The classification of energy states based on entropic energy is summarized in Table 1.

The TCI objectively defines exergy, entropic energy, and energy states based on the reversible measurements of work and heat with respect to the ARS. We note that the HCF, with its assumption of an absolute-zero ambient surrounding, does not recognize heat or entropic energy as fundamental, and it only recognizes the mechanical energy states.

### 3.4. Microstates, Macrostates, and Information

Up to this point, the TCI has only addressed energy states. A system is more than just its energy state, however. The configuration of a system’s parts defines the system’s microstate, which is generally just one of a vast number of possibilities. Whereas the energy state is defined by perfect reversible measurement results (Figure 1), a system’s thermocontextual microstate configuration is defined by information from perfect observation. Perfect observation provides complete information by a perfect ambient observer with resolution defined by the ambient temperature.

**Figure 4:**
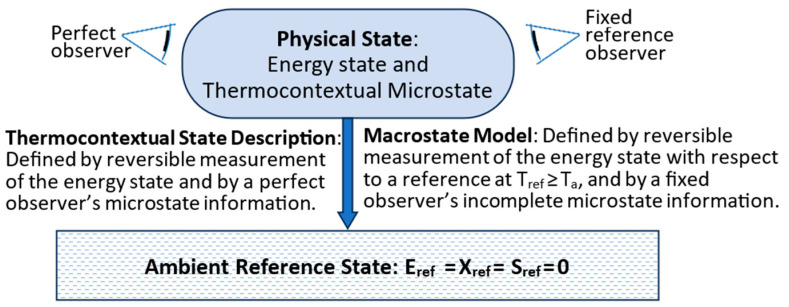
Thermocontextual state description and macrostate model. The thermocontextual state is completely defined by its energy-state measurement with respect to the ambient reference and by its microstate description by a perfect ambient observer. The macrostate model, in contrast, is defined with respect to a reference at a fixed reference temperature, T_ref_ ≥ T_a_, and by a generally imperfect observer with incomplete information.

Figure 4 (left side) illustrates the relationships among a system’s physical state, its energy state, its thermocontextual microstate, and its complete thermocontextual state description. The thermocontextual state description completely defines the system’s physical state.

The right side shows the same system’s macrostate model description by an imperfect observer. An imperfect observer’s information can be limited by imperfect measurement or random changes. In either case, the imperfect observer’s information is generally incomplete.

We can describe the macrostate model’s incomplete description statistically as a probability distribution by the following:

(24) $$P_{MS}=\left[P_{ms,1},P_{ms,2},\ldots P_{ms,N_{obs}}\right].$$

where the macrostate is based on the reference observer’s generally incomplete information. N_obs_ is the number of microstates resolvable by the observer, and the P_ms,i_ are the Bayesian probabilities [4,15] describing the reference observer’s expectations that the system’s observable microstate is ms,i. The macrostate model is a superposition of the reference observer’s microstate expectations.

We define configurational entropy as a macrostate property describing the imprecision of the observer’s macrostate model description. It is defined by the following:

(25) $$\sigma_{c}\equiv -\sum_{i=1}^{N_{obs}}P_{i}\ln\left(P_{i}\right),$$

where the sum is over the system’s observable microstates. The P_i_s are the macrostate model’s Bayesian probabilities, which are assigned by the fixed-reference observer based on its fixed information. The configurational entropy is equal to statistical entropy, but in contrast to (5), σ_c_ is summed over the number of resolvable microstates (N_obs_). It is explicitly based on the reference observer’s available information and the resolution of microstates.

To illustrate and contrast the macrostate and the physical state that it describes, we consider a fair and random coin flip. The macrostate model transitions from a single initial known state of either heads or tails, with N = 1 and **P_MS_** = [1], to a mixed macrostate following the coin flip, with N = 2 and **P_MS_** = [½, ½]. The macrostate model following the coin flip describes equal and superposed expectations for heads or tails configurations, and its configurational entropy increases from zero to *ln*(2).

Following the coin flip, the coin has a definite but unknown microstate, and its physical state is statistically described as **P_PS_** = [½, ½]. It is equal to the macrostate’s probability distribution, **P_MS_**. However, whereas the macrostate model’s probabilities are expectation-based Bayesian probabilities, the physical state’s unknown microstate’s probabilities are frequentist probabilities [16]. Frequentist probabilities are defined by the statistics of the repeated physical measurements of, or following, a random transition.

The coin’s physical state may be unknown, but it exists as a single definite microstate. Its actual microstate configuration is represented as follows:

(26) $$P_{msa}=\left[P_{ms,a}=1,P_{ms,i\neq ms,a}=0\right],$$

where **msa** is the physical state’s actual microstate configuration and ‘ms,a’ indicates the actual but unknown microstate of heads or tails. We would like to have a measure of the “distance” between the macrostate model and the system’s actual microstate.

The Kullback–Leibler divergence (D_KL_) [17] was introduced to provide a measure of the information gap between two probability distributions. The D_KL_ divergence is defined by the following:

(27) $$D_{KL}(P_{1}||P_{2})\equiv \sum_{i=1}^{N}P_{1,i}ln\left(\frac{P_{1,i}}{P_{2,i}}\right).$$

The D_KL_ information gap describes the relative entropy of state 1 based on the macrostate model 2. We can apply (27) to the information gap between the reference observer’s Bayesian macrostate model, **P_MS_** (24), as **P_2_** and the system’s actual but unknown microstate, **P_msa_** (26), as **P_1_**. Substituting (26) and (24) for P_1_ and P_2_, the D_KL_ divergence collapses to the following:

(28) $$D_{KL}(P_{msa}||P_{MS})=ln\left(\frac{1}{P_{ms,a}}\right).$$

Equation (28) is our measure of the macrostate model’s accuracy. P_ms,a_ is the reference observer’s Bayesian expectation probability for the actual observable, but unknown, microstate ‘a’. The D_KL_ information gap ranges from zero for P_ms,a_ equal to one, representing complete knowledge of the actual observable microstate configuration ‘a’, to infinity for P_ms,a_ equal to zero, representing complete misinformation.

Equation (28) shows that as P_ms,a_ approaches one hundred percent, the D_KL_ information gap approaches zero, and vice versa. As P_ms,a_ approaches one, the macrostate model’s entropy (25) also approaches zero, representing the reference observer’s complete description of the observable microstate. These relationships are summarized by:

(29) $$\left(D_{KL}\to 0\right)⟺\left(P_{ms,a}\to 1\right)⟹\left(\sigma_{c}\to 0\right).$$

A zero D_KL_ implies a zero configurational entropy, but a zero configurational entropy does not imply a zero-information gap. A macrostate entropy of zero could have P_ms,j_ equal to one for some microstate j not corresponding to the actual microstate, and P_ms,a_ would equal zero, representing an infinite information gap. Configurational entropy describes a macrostate model’s precision, but precision does not mean accuracy. The D_KL_ information gap, not configurational entropy, describes a macrostate model’s accuracy and a reference observer’s uncertainty of a system’s actual microstate configuration.

## 4. Transitions

We now shift focus from states to a system’s transitions between states. A transition is described by transactional properties (Appendix A). A transition’s decline in exergy, for example, is described by the following:

(30) $$\overset{ˇ}{X}=\hat{Q}+{\hat{w}}_{a}=k_{B}T_{a}{\hat{\sigma }}_{q}+{\hat{w}}_{a}.$$

where $\overset{ˇ}{X}$ is the transactional decline in a system’s exergy per transition; ${\hat{w}}_{a}$ is the output of ambient work to the ambient surroundings; and $\hat{Q}$ and ${\hat{\sigma }}_{q}$ are the irreversible productions of entropic energy and thermal entropy by dissipation. Equation (30) follows from (6), (19)–(21), and the conservation of energy.

### 4.1. Updated Postulates of Transitions

Postulates Four and Five, as originally proposed in [6,14], are limited to energy-state transitions with respect to fixed ambient surroundings. A thermocontextual state transition, however, can involve changes in the ambient temperature, random changes in the system’s microstate configuration, or changes in the observer’s information due to observation or memory loss. We need to describe transitions with respect to a fixed reference state and define a fixed information state.

By convention, we define a fixed reference’s temperature by a system’s initial ambient temperature, and we define a fixed information state by perfect ambient measurement of the initial state. A fixed-reference observer’s information on a system is, therefore, complete at time zero, and the information gap is zero. However, as described above, transitions can take an initially complete description and make it incomplete. TCI’s postulates of transition need to be updated to account for these possibilities.

Before updating TCI’s Postulates Four and Five, however, we need to introduce two new macrostate model properties (Appendix A). Configurational energy is defined here as the portion of a system’s exergy that is inaccessible to the reference observer for work. A system’s exergy could be inaccessible either due to incomplete information on its observable microstates, or due to an elevated reference temperature of measurement, T_ref_ > T_a_, or both. Configurational energy and its mechanical and thermal components are defined by the following:

(31) $$C=C_{m}+C_{q}\equiv k_{B}T_{ref}D_{KL}+\int_{T_{a}}^{T_{ref}}\frac{\left(T-T_{a}\right)}{T}C_{v}dT$$

(32) $$C=k_{B}T_{ref}D_{KL}+C_{v}\left(T_{ref}-T_{a}-T_{a}\ln\left(T_{ref}\right)+T_{a}\ln\left(T_{a}\right)\right).$$

**Figure 5:**
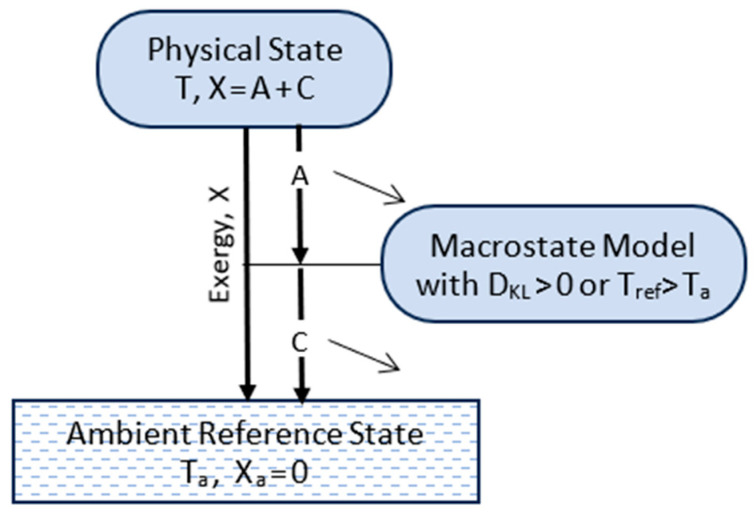
Accessibility and configurational energy. Configurational energy C is unavailable to the reference observer for work due to T_ref_ > T_a_ or due to incomplete microstate information. Accessibility A is the balance of exergy X that is reversibly available to the reference observer for work.

C_q_ describes the thermal exergy that is not available to the observer due to its reference temperature of measurement being greater than the ambient temperature (Figure 5). C_m_ describes the mechanical exergy that is not available to the observer due to incomplete information on the thermocontextual microstate, as described by a positive D_LK_ information gap. C_m_ = k_B_T_ref_D_KL_ is analogous to entropic energy, Q = k_B_T_a_σ_q_. If D_LK_ is zero, the observer has complete information on the mechanical microstate configuration, and C_m_ equals zero.

The balance of exergy is accessibility. Accessibility is the portion of a system’s exergy that is accessible to the observer for work at the fixed reference temperature. The partitioning of exergy into accessibility and configurational energy is illustrated in Figure 5.

Accessibility is defined by the following:

(33) $$A\equiv X-C=\left(X_{m}-C_{m}\right)+\left(X_{q}-C_{q}\right)$$

(34) $$A={(X}_{m}-k_{B}T_{ref}D_{KL})+X_{q}-C_{v}\left(T_{ref}-T_{a}-T_{a}\ln\left(T_{ref}\right)+T_{a}\ln\left(T_{a}\right)\right)$$

(35) $$A={(X}_{m}-k_{B}T_{ref}D_{KL})+C_{v}\left(T-T_{ref}-T_{a}\ln\left(\frac{T}{T_{ref}}\right)\right),$$

where exergy, accessibility, and configurational energy are all broken into their mechanical and thermal components (32), and thermal exergy X_q_ is expanded by the integration of (16) from the ambient temperature to the system temperature. Equation (34) shows that if T_a_ = T_ref_, and if, in addition, its actual configuration is precisely known (D_KL_ = 0), then A = X_m_ + X_q_ = X, and the exergy is entirely accessible at time zero. This describes a system’s state at time zero. If the ambient temperature declines or randomness increases the information gap, then the exergy is only partially accessible for work (A < X).

With accessibility defined, we can now update Postulate Four:

**Postulate 4:**  *(Stability of states—The Minimum Accessibility Principle): A state with positive accessible exergy is unstable and has a potential to spontaneously transition to states with lower accessible exergy.*

Postulate Four updates Postulate Four from [6] and [14] by replacing exergy in the original version with accessibility. Whereas exergy is defined by a perfect all-knowing ambient observer, accessibility is defined by a fixed-reference observer based on its actual information. Accessibility extends the applications of Postulate Four to describe transitions involving changes in a system’s physical surroundings, or to changes in the information gap. Postulate Four states that a state has a spontaneous potential to transition to a state of lower accessible exergy and higher stability. Maximum stability is defined by equilibrium with the ambient surroundings, with zero exergy and minimum accessibility.

Before introducing the updated Postulate Five, we need to define utilization as a transactional property of transitions:

(36) $$\hat{\upsilon }\equiv {\hat{w}}_{ref}+{\hat{A}}_{int}.$$

Utilization is the sum of external work, ${\hat{w}}_{ref}$, plus internal work, ${\hat{A}}_{int}$, per unit transition. ${\hat{w}}_{ref}$ is the work plus accessible energy output to the fixed reference, and ${\hat{A}}_{int}$ is the internal work of increasing the system’s accessible exergy change per transition.

With the macrostate model and transactional properties defined, we can now update TCI’s original Postulate Five. Postulate Five is updated here by replacing the original dimensionless “utilization efficiency” [14] with transactional utilization (36):

**Postulate Five:**  *(Stability of transitions—The Maximum Utilization Principle): A transition seeks to maximize its utilization (work output plus accessibility increase).*

Whereas Postulate Four addresses the stability of states, Postulate Five addresses the stability of transitions. The updated Postulate Five states that the transition with the highest utilization is the most stable.

### 4.2. Transitions, Dissipation, and Dispersion

From Equations (30)–(33), we can express a general system’s loss in accessibility by the following:

(37) $$\overset{ˇ}{A}=k_{B}T_{a}{\hat{\sigma }}_{q}+{k_{B}T_{ref}\hat{D}}_{KL}+{\hat{w}}_{ref}.$$

where $\overset{ˇ}{A}$ is the decline in accessible exergy due to increases in thermal entropy (dissipation) and D_LK_ information gap (dispersion) plus the output of work to the fixed reference. Equation (37) generalizes (30) to address changes in the system, in the ambient surroundings, or in the reference observer’s information gap.

**Figure 6:**
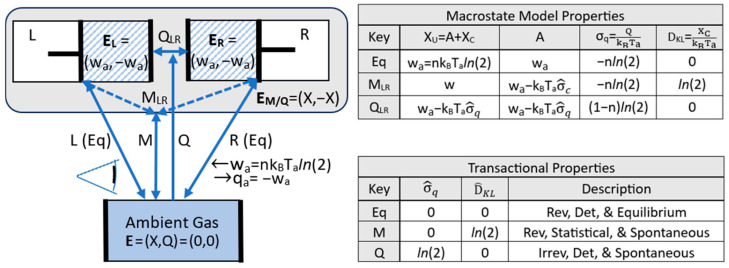
Quasistatic transition types. The figure illustrates the three types of transitions from ambient gas to compressed gas by the application of ambient work, w_a_. The transitions are quasistatic, with no frictional losses. The equilibrium transitions, L and R, reversibly and deterministically compress the gas from either the left or right side and produce a definite and known microstate, L or R. Transition M is reversible but statistical, and it produces a mixed macrostate M_LR_, with a single definite but unknown microstate L or R. Transition Q irreversibly but deterministically compresses the gas and produces a single thermalized microstate, Q_LR_. The macrostate model and transactional properties for each are shown in the tables on the right side.

The relationship between accessibility and these various changes can be clarified by considering the configurational energy state from Figure 2 (c). The reversible work to create the energy state in Figure 2 (c) could just as well have compressed the gas from the left or the right, as shown by the two configurations in Figure 6. The transitions in Figure 6 are all at ambient temperature, but they involve an increase in the pressure. The number of thermocontextual microstate configurations resolvable by the system’s ambient observer is N_obs_ = 2. (An ambient observer cannot resolve gas particles). The compressed gases in each of the two configurations share the same energy state, and they are degenerate. They have the same exergy, equal to the work w_a_ = nk_B_T_a_*ln*(2) of reversibly compressing an n-particle ambient gas to one-half volume.

Figure 6 illustrates three basic types of quasistatic transitions for the compression of the ambient gas to one-half volume, as described by a fixed observer. A quasistatic transition proceeds at a negligible rate so that dissipation due to friction can be neglected. The transitions all have the same reversible exchanges of ambient work and ambient heat, but they differ in their exergy and information changes, as shown by the differences in ${\hat{\sigma }}_{q}$ and ${\hat{D}}_{KL}$ in the figure’s lower table. The upper table shows the exergy, accessibility, entropy, and D_KL_ information gap for the system following each transition.

Transitions L and R utilize the work of input to reversibly and deterministically compress the gas from the left or right side to produce a definite configuration. The L and R configurations are known, and so their D_KL_ information gaps are zero. There is zero loss of information, and this is the definition of determinism. The work of compression is reversibly stored and fully accessible, and so X = A = w_a_. From (6) and (19), the thermal entropy is σ_q_ = Q/k_B_T_a_ = −nl*n*(2). These results are summarized in the upper table (top row). As summarized in the lower table (top row), there is no production of thermal entropy, no loss of information, and no increase in the D_KL_ information gap. Zero production of thermal entropy means no dissipation, and this is the definition of reversibility. The quasistatic transition from ambient state to L or R is reversible and deterministic, and this defines an equilibrium transition for a mechanical time-dependent state.

Transition M takes the work of input and produces a mixed macrostate, M_LR_, with a definite but unknown microstate configuration of L or R. The fixed observer’s information on the initial gas is complete, but following the gas’s compression, it is incomplete. Transition M is statistical, and it produces configurational entropy ${\hat{\sigma }}_{c}=ln(2)$, based on (25) and two equal-probability configurations. The production of configurational entropy and the contrapositive of Equation (29) indicate an increase in the D_KL_ information gap, and from (37), a loss of accessible exergy. The loss of accessibility is related to the loss of information; without knowing the configuration of the compressed gas, its exergy cannot be accessed for work with perfect efficiency and certainty. From Postulate Four, the loss of accessibility means that the process of producing configurational entropy and increasing the information gap is spontaneous.

The mixed macrostate’s definite configuration is unknown, but it is definite. By Postulate Three, it is not hidden; it is knowable to a perfect ambient observer; and the transition, therefore, preserves exergy. There is no irreversible production of thermal entropy, dissipation, or thermalization, and the transition is, therefore, reversible. The M transition to the mixed macrostate is statistical and spontaneous, but it is also reversible, as summarized in the lower table in Figure 6.

Transition Q applies the work of input to the zero-entropy ARS and transitions it to a thermalized configuration, Q_LR_. The Q_LR_ configuration exists as a “pure” thermalized microstate configuration, with positive thermal entropy and L and R as uninstantiated potentialities. The transition is from a single ambient configuration to a single thermalized configuration, and this makes it deterministic. Determinism means that there is no increase in uncertainty or D_KL_ information gap. Thermalization, however, means that the transition produces thermal entropy, and it is irreversible.

The transition from the zero-entropy ambient microstate to the thermalized microstate Q_LR_ in Figure 6 reflects the irreversible production of thermal entropy (${\hat{\sigma }}_{q}=ln(2)$). From (37), the production of thermal entropy results in the irreversible loss of exergy and also a loss of accessible exergy. With a loss of accessibility, the transition Q is deterministic, irreversible, and spontaneous, as summarized in the lower table in Figure 6.

The transactional properties of the three quasistatic transitions are summarized in the lower table in Figure 6. The quasistatic Eq transitions are reversible, deterministic, and equilibrium. The M and Q transitions are both spontaneous, with a loss of accessible exergy. For the M transition, the loss of accessibility is due to the loss of information by dispersion, and for the Q transition, the loss of accessibility is due to the production of thermal entropy and the dissipation of exergy. Eq, M, and Q transitions are three fundamentally distinct types of quasistatic transitions. An actual finite-rate transition is not quasistatic, of course, and it includes dissipation due to frictional forces.

An immediate consequence of (37) and Postulate Four is that a system has a spontaneous potential to dissipate exergy, to disperse information, or both. The dissipation of exergy by irreversible entropy production is precisely the subject of the Second Law of thermodynamics. The Second Law and TCI’s original Postulate Four (minimum exergy), are both about Q-type transitions and the irreversible dissipation of exergy. In contrast, the spontaneous dispersion of information is precisely the subject of MaxEnt [4,9]. MaxEnt is about M-type transitions to mixed macrostates, and the spontaneous and statistical, but reversible, dispersion of microstate configurations. Whereas dissipation produces thermal entropy, dispersion increases the D_KL_ information gap.

An M-type transition increases a system’s information gap with respect to a fixed-reference observer, but a system can only change until it reaches equilibrium with its ambient surroundings. At equilibrium, the mixed macrostate can exist in any one of multiple possible configurations, but they all have zero exergy, and the reference observer has zero information on the system’s actual microstate. With zero information, the reference observer assigns all the microstates equal unbiased expectations of 1/N, and the D_KL_ information gap (27) immediately reduces to the following:

(38) $$D_{KL}(P_{\mu Sa}||[\frac{1}{N},\frac{1}{N},\ldots \frac{1}{N}])=ln\left(N\right)=\sigma_{max}.$$

At equilibrium, the ambient reference observer has zero information, and the D_KL_ information gap reduces to the maximum theoretical configurational entropy, regardless of which microstate configuration (26) the physical state transitions to. This precisely expresses MaxEnt.

The implications of Postulate Four are expressed by three corollaries:

**Corollary 4-1.**  *The Second Law of Thermodynamics—thermodynamic entropy is irreversibly produced and can never decline.*

**Corollary 4-2.**  *The Minimum Exergy Principle (TCI’s original Postulate Four)—given a fixed ambient temperature, exergy is irreversibly dissipated to ambient heat.*

**Corollary 4-3.**  *MaxEnt−a non-dissipative system with fixed ambient surroundings has a spontaneous potential for dispersion to increase the D_KL_ information gap and to drive configurational entropy to its maximum.*

Corollaries 4-1 and 4-2 describe the irreversible dissipation of Q-type transitions. Corollary 4-1 (the Second Law) states that thermodynamic entropy irreversibly increases, and Corollary 4-2 states that exergy is minimized. Corollary 4-3 describes M-type transitions, which are statistical and spontaneous but reversible, in principle. It states that spontaneous dispersion drives an increase in the D_KL_ information gap, but only up to the point of equilibrium and maximum configurational entropy.

### 4.3. Efficiency and Refinement

Postulate Four addresses the stability of states. In this section, we apply Postulate Five to address the stability of transitions and processes, with both fixed and changing environments. Postulate Five states that the most stable transition has the highest utilization. Equations (36) and (37) showed that a transition’s utilization is related to (1) work output, (2) thermal entropy production (dissipation), (3) ambient temperature, and (4) the increasing D_KL_ information gap (dispersion). We introduce four corollaries of Postulate Five addressing the stability of transitions based on each of these factors.

Corollaries 5-1 and 5-2 apply to stationary dissipative processes with fixed ambient temperature and information gap. Stationary does not mean static, however, and a stationary dissipative system is not a state. A stationary system’s time-averaged properties of state are constant, but they can fluctuate around fixed averages, and dissipative systems have positive flows of components and energy.

**Figure 7:**
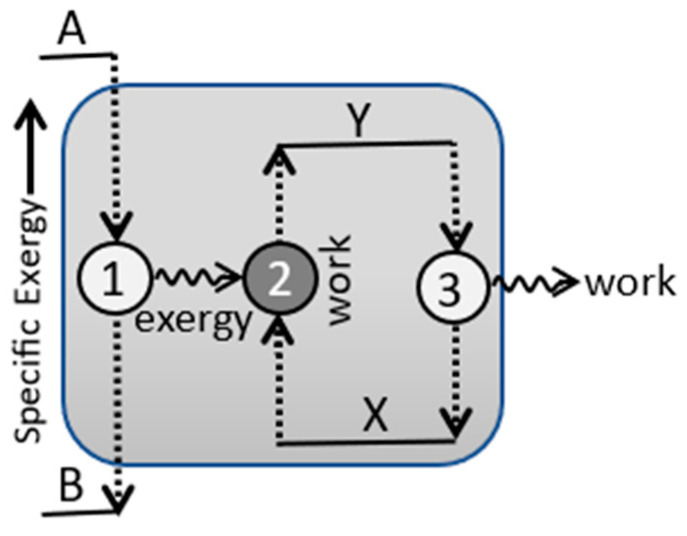
Dissipative network diagram for the reactions A + X → B + Y and Y → X. States are represented as horizontal lines with specific exergy per unit of component. Transitions between states are represented by dotted lines and numbered nodes. Transition 1 extracts exergy from input A as it transitions to output B. Exergy is transferred to the coupled transition node 2, which does the internal work of converting X to the higher-exergy state Y. Transition 3 takes state Y back to X and uses the extracted exergy for external work on the surroundings.

Crecraft [14] referred to stationary dissipative systems as homeostates. They are also referred to as nonequilibrium stationary states (NESS) by Ribó and Hochberg [18,19], and as flow networks by Robert Nivens et al. [20,21]. Flow networks, NESS, and homeostates all describe a stationary dissipative system as a network of transitions. Figure 7 illustrates a homeostate as a network of directed component links connecting irreversible transition nodes, all at a fixed ambient temperature. Components have well-defined state properties, but as they transition, their exergy is partially dissipated to heat and partially output as work or exergy to the environment or to other nodes within the network.

A dissipative system’s net efficiency is equal to the ratio of external work output to exergy input, and it cannot exceed one hundred percent. For a network of dissipative nodes, however, the system’s total work includes the internal work on other network nodes. A stationary system’s total efficiency is defined by the ratio of total work to exergy input. Autocatalytic loops can recycle energy internally and yield total work efficiencies exceeding one hundred percent [14]. It can be seen that in the limit of zero dissipation, the total efficiency of the dissipative network in Figure 7 approaches two hundred percent.

**Figure 8:**
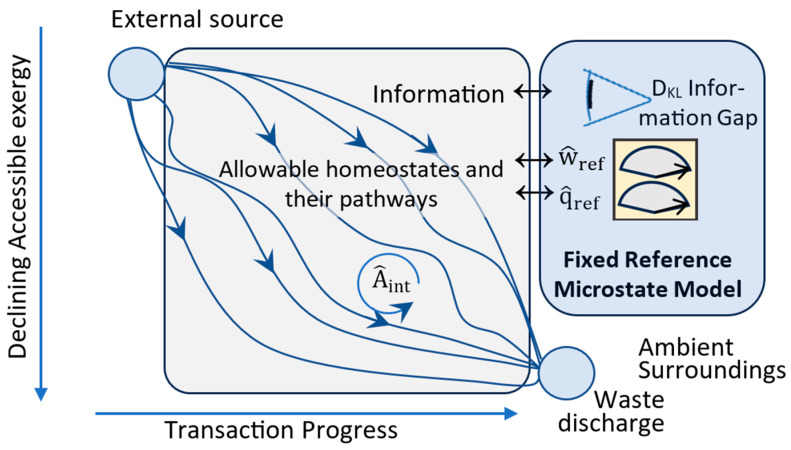
Stationary dissipative processes and homeostates. Each path represents a homeostate and the process of transitioning source components to the ambient surroundings. Observation reduces the observer’s information gap by revealing information on the network nodes, transitions, and the internal work of increasing the system’s accessibility. An external device measures the outputs of reference work (mechanical work plus accessible energy) and heat to the fixed reference. Perfect observation and measurement are in the quasistatic limit of zero frictional losses of exergy and information.

The non-linearity of far-from-equilibrium systems allows for multiple homeostates and dissipative processes. Postulate Five addresses the relative stabilities of competing processes and homeostates. Figure 8 illustrates the application of Postulate Five to a far-from-equilibrium system. Each path in the figure represents a different process and a distinct homeostate that can exist for a given stationary environment. The dissipative processes and homeostates can have different network structures, flow rates, and efficiencies. Figure 8 also shows the observation and measurement of the homeostate transitions by a reference observer and its measurement device at the model’s reference temperature.

Each path in Figure 8 represents a stationary dissipative process and homeostate with high-accessibility inputs and low-accessibility wastes. Each homeostate path generally has multiple transitions and intermediate states. Internal work is the work by nodes on components within the dissipative network (Figure 7). Internal work (${\hat{A}}_{int}$) is based on observable component flows and accessibility increases within the network. Postulate Five calls for a stationary dissipative system to increase its utilization by increasing its internal plus external work per unit of transition.

A system can increase its utilization in two ways: (1) by growth (or replication), or (2) by increasing efficiency. When resources are available, growth happens. Fires spread, and bacteria multiply. This is expressed by Corollary 5-1:

**Corollary 5-1.**  *The Growth Principle: A dissipative system expands to its stationary environment’s carrying capacity.*

As a system expands, it increases its rates of exergy input, dissipation, and work. A larger system always has a higher rate of work compared to a smaller system. A larger stationary system is consequently always more stable, creating a spontaneous drive for growth.

There is a limit to sustainable growth, however, due to finite resources. A mature ecosystem, for example, is a stationary homeostate in which its various species and the environment are in dynamic balance. A mature ecosystem at its environment’s carrying capacity cannot expand. The system can continue to evolve, however, by increasing its total efficiency and internal accessibility. This is expressed as Corollary 5-2:

**Corollary 5-2.**  *The maximum efficiency principle (TCI’s original Postulate Five): A dissipative system with a stationary environment spontaneously transitions to a stationary process of higher total efficiency.*

Total efficiency is defined by the following [14]:

(39) $$\Xi =\frac{\hat{\upsilon }}{{\hat{w}}_{ref,in}},$$

where $\hat{\upsilon }$ is the utilization (36), equal to the sum of reference work output plus internal work, and ${\hat{w}}_{ref,in}$ is the input of reference work (mechanical work plus accessible energy).

The next corollary is thermal refinement. It applies to a fixed-temperature, non-dissipating (fixed mechanical exergy), and non-dispersive (fixed D_KL_) system with changing ambient surroundings.

Differentiating (34), the change in accessibility with ambient temperature is given by the following:

(40) $$\frac{dA}{dT_{a}}=C_{v}ln\left(\frac{T_{ref}}{T}\right).$$

If the system and its ambient surroundings are initially in thermal equilibrium, then the initial ambient temperature and system temperature, T, are equal. From (7), the fixed reference temperature and system temperature are also equal, and from (40), $\frac{dA}{dT_{a}}$ equals zero. For a system initially in thermal equilibrium, a change in the ambient temperature has no effect on accessibility, and from Postulate Five, there is no potential for its change.

If the system and ambient temperature are not initially equilibrated, then from (7), there exists a fixed reference temperature and ambient temperature less than the system temperature, and $\frac{dA}{dT_{a}}$ is less than zero. If a system is not thermally equilibrated with its ambient surroundings, then from (40), a decline in the ambient temperature increases the system’s accessibility. From Postulate Five, the ambient temperature, therefore, has the potential to spontaneously decline.

The TCI recognizes thermal refinement as a special-case transition that increases a system’s accessibility by lowering the system’s ambient temperature. Thermal refinement is a special case of Postulate Five, as expressed by the following:

**Corollary 5-3.**  *Thermal Refinement: For a non-equilibrium system with positive thermal energy, its ambient temperature has a spontaneous potential to decline, resulting in an increase in a fixed-reference observer’s access to the system’s thermal exergy.*

Corollary 5-3 provides a drive for a dissipative system to discharge waste heat to a lower-temperature environment, thereby increasing the accessibility of a system’s thermal energy for work.

Thermal refinement does more than extract exergy from entropic energy, however; it also derandomizes a thermalized system and instantiates one of its microstate potentialities. Interaction with the newly cooled surroundings triggers the random selection of a thermalized microstate potentiality and instantiates it as a definite and observable microstate configuration with positive accessible energy.

The fourth and final corollary is the configurational refinement of a state with a fixed ambient temperature, fixed energy, and no dissipation. As noted in Section 3.3, an energy state can have any of the multiple different microstate possibilities, all consistent with the system’s physical and boundary constraints. The reference observer’s knowledge of the energy state’s macrostate is expressed as a probability distribution over the available microstate potentialities (24). Each probability expresses the observer’s expectation that a given microstate potentiality will be instantiated upon measurement.

The reference observer’s macrostate model changes with changes in information by observations (or memory loss). As shown by (37), for a non-dissipative system with ${\hat{\sigma }}_{q}=0$, a narrowing D_KL_ information gap (28) provides an external observer or agent greater access to the system’s exergy for work. This is expressed by Corollary 5-4:

**Corollary 5-4.**  *Configurational Refinement: An external observer or agent has a spontaneous potential to reduce its D_KL_ information gap with a positive-exergy system to increase its access to the system’s exergy for work.*

Whereas Corollary 4-3 drives a system to increase its configurational entropy, Corollary 5-4 provides an important counter to this by driving an external agent to spontaneously narrow its information gap. This increases the system’s accessibility, in compliance with Postulate Five. Configurational refinement means a narrowing information gap, and this is the informational analog of thermal refinement.

The mandate of Postulate Five is to increase a transition’s utilization (36). Corollaries 5-3 and 5-4 provide the drive to reduce uncertainty through thermal and configurational refinement and to increase a system’s accessible exergy. Corollaries 5-1 and 5-2 maximize utilization of that accessible energy first by expanding to a stationary environment’s carrying capacity, and then by maximizing efficiency.

## 5. Applications

This section discusses the applications of Postulates Four (minimum accessibility of state) and Five (maximum utilization of process) and their corollaries. Postulate Four’s Corollary 4-1 is the Second Law of thermodynamics. The Second Law has been thoroughly vetted, and it needs no further discussion. Corollary 4-2 was introduced in [6] as the Minimum Exergy Principle and as TCI’s original Postulate Four. It is closely related to the Second Law of thermodynamics, and it states that exergy is irreversibly dissipated.

Corollary 4-3 recognizes MaxEnt as a special case of Postulate Four. The TCI recognizes configurational entropy and MaxEnt as distinct from thermodynamic entropy and the Second Law of thermodynamics. The Second Law expresses irreversible dissipation and the production of thermodynamic entropy; MaxEnt expresses the spontaneous dispersion of information and the production of configurational entropy. Section 5.1 applies MaxEnt to the double-slit experiment to explain the well-documented but previously unexplained results, including random symmetry breaking.

Postulate Five’s Corollary 5-1 (growth principle) simply says that given an opportunity, a dissipative system will expand to the carrying capacity of its environment. Growth increases the system’s rate of work. The spontaneous potential for growth is a widely recognized phenomenon, but it has commonly been misinterpreted as a consequence of a proposed principle of maximizing the rate of entropy production [22,23,24]. The rates of entropy production and work are both correlated with size and transition rate, but the drive for growth is driven by the increase in work rate, not by the rate of thermal entropy production. Whirlpools, for example, have a higher rate of total internal work but a lower rate of entropy production [14]. Their spontaneous formation provides an important counterexample of the maximum entropy production principle.

Corollary 5-2 was introduced in [14] as TCI’s original Postulate Five, the maximum efficiency principle. Given a fixed environment, Corollary 5-2 preferentially selects higher-efficiency dissipative processes. The article illustrated the principle with numerous applications based on well-documented examples of spontaneous self-organization.

Given a stationary environment, Corollaries 5-1 and 5-2 provide two paths for a dissipative system to increase its utilization. When resources are available, systems expand to their environment’s carrying capacity. When resources become constrained, the drive for continued growth leads to competition for limited resources, and at some point, continued growth becomes unsustainable. The system can continue to increase its rate of work, however, by increasing efficiency through cooperation and increasing the organization of dissipative networks. Corollary 5-2 defines the arrow of increasing functional complexity.

Corollary 5-3 addresses thermal refinement. It states that a system has a spontaneous potential to reduce the temperature of heat discharge to increase the accessibility of its energy for work. For a dissipative system, thermal refinement slows or even reverses the decline in exergy. If the 2.5 K cosmic microwave background (CMB) temperature is taken as the ambient temperature of the universe, then we can recognize cosmic expansion, which reduces the CMB temperature, as a mechanism of thermal refinement, in compliance with Corollary 5-3. As the ambient temperature falls, thermal exergy increases.

Thermal refinement also leads to the instantiations of new microstate configurations. During the early universe, when the universe cooled to about 2 × 10^12^ K, the universe became unstable with respect to both baryons and antibaryons, but the transition almost exclusively produced baryons [25]. The asymmetrical transition to baryons and matter over antimatter is referred to as baryogenesis, and it is considered one of the outstanding problems of modern physics. Crecraft showed that Postulate Five promotes the synchronization of parallel transitions to reduce dissipation and increase work output [14]. The synchronized instantiation of either baryons or antibaryons over a mixture of the two would reduce dissipation by mutual annihilation, and it would increase the work of particle creation, in compliance with Postulate Five. The instantiation of matter over antimatter may simply have been a spontaneous synchronized process of random symmetry breaking to increase the transition’s efficiency.

Corollary 5-4 can explain the spontaneous decline in the configurational entropy of the SARS-CoV-2 virus documented by Vopson and Lepadatu [1]. The virus has the role of an external agent, and the virus’s target cells are the system. The change in the virus’s RNA reflects a closing of the D_KL_ information gap between the information encoded in the virus’s RNA and the information encoded in the nucleotides of the virus’s target cells. Narrowing the information gap increases the virus’s access to its target’s energy. In the case of the virus, the narrowing information gap is achieved through random mutations and selection. Corollary 5-4 provides a selection criterion for the virus to favor mutations that enable it to access its target’s energy and to increase its work of reproduction. From (29), the decline in the D_KL_ information gap means a decline in the configurational and information entropy, as described by Vopson and Lepadatu’s original law of infodynamics.

We note that Corollary 5-4 does not apply to the decline in entropy for Vopson and Lepadatu’s first example with the magnetic storage device. Corollary 5-4 applies to non-dissipative M-type transitions, but the thermalization of the magnetic storage medium is a Q-type transition. Increasing thermalization reduces a perfect observer’s resolution and the number of resolvable microstates. From (25), this reduces the configurational (information) entropy. However, thermalization is a dissipative process, and it leads to spontaneous decreases in the system’s exergy and accessibility. The magnetic storage device’s entropy decline in their first example is instead a consequence of Corollary 4-1 (Second Law of thermodynamics).

Corollaries 5-4 and 4-3 together underpin Bayesian statistical modeling and analysis, which has become a powerful tool for analyzing complex systems [26]. E. T. Jaynes showed that maximizing entropy produces an unbiased best-fit model based on available information [4]. From Corollary 4-3, a system naturally maximizes the configurational entropy of its macrostate model, and this is the first key for the application of Bayesian statistical analysis. From Corollary 5-4, an external agent (i.e., researcher) has a spontaneous drive to improve its macrostate model’s accuracy, as quantified by minimizing the D_KL_ information gap. This is the second key for Bayesian analysis. By successively updating the system’s macrostate model with new information and allowing it to maximize its entropy, an observer can progressively improve its macrostate model’s accuracy of a complex dissipative system.

Recent applications of Bayesian statistical modeling include problems in astrophysics [27], rapid medical diagnostics [28,29], thermodynamic computing [30,31], artificial intelligence and machine learning [32,33], ecological modeling [34], macroeconomics [35], imaging theory and applications [36,37,38], network analysis [20,21,39], and plasma science [40].

A final application of Corollary 5-4 is discussed in Section 5.2. It illustrates a simple model for the abiogenic origin of self-replicating nucleotides.

### 5.1. MaxEnt and the Double-Slit Experiment

In the double-slit experiment, a quantum of energy is emitted from a source as a particle, and it is detected as a particle by its point-like impact on a detector screen [41]. If a partition with double slits is placed between the source and detector screen, the accumulated impacts display an interference pattern, characteristic of waves, even when the particles are transmitted one at a time. If a “which-slit detector” (WSD) is inserted behind the slits and activated, it interacts with the quanta, and the wave interference pattern disappears. Richard Feynman famously described the double-slit experiment as the “*only* mystery”, “which has in it the heart of quantum mechanics” [42]. The TCI and the following discussion, updated from [43], offer an important new insight into this behavior.

**Figure 9:**
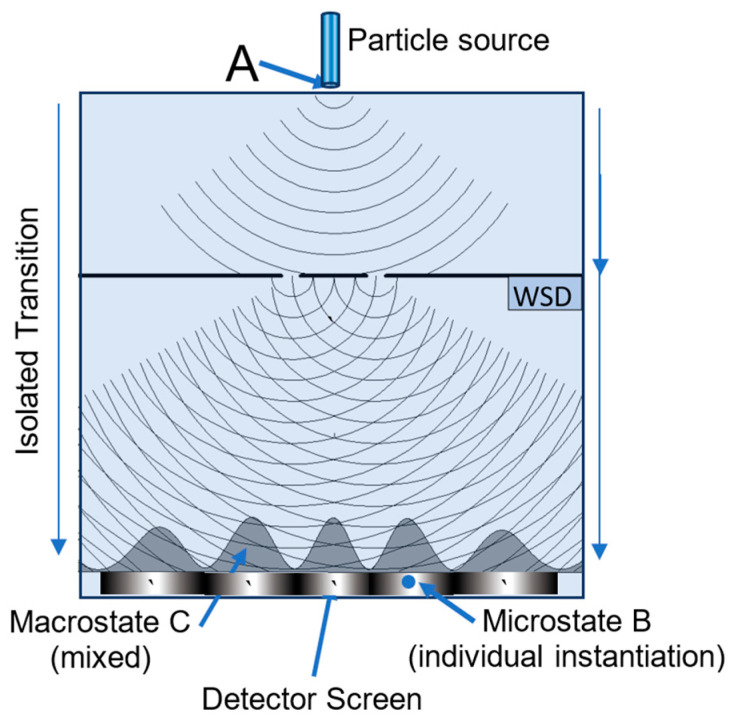
Double-slit experiment. The microstate configurations are defined by the resolution of the detector screen. If the which-switch detector (WSD) is disabled, an individual transition passes through the double slits symmetrically and randomly instantiates an impact and definite configuration on the detector screen (state B). Multiple transitions generate a statistically mixed macrostate C, represented by a probability distribution of instantiated microstates. With the WSD activated, the particle passes asymmetrically through one slit or the other, and the interference pattern for C changes to a single broad peak.

Figure 9 illustrates the double-slit experiment. A quantum of energy is emitted as a definite particle, and it transitions to a mixed macrostate comprising one of many possible point-like configurations, each recorded as an impact on a detector screen (e.g., point B). Between the points of emission and detection, the particle is in an M-type transition from a definite microstate configuration to a mixed macrostate of higher configurational entropy. With the WSD deactivated, multiple transitions generate a statistically mixed macrostate with a wave-like interference pattern, represented by the probability distribution profile C in Figure 9. The profile represents the cumulative effect of individual transitions and the record of multiple instantiations of definite microstate configurations.

With the WSD activated, a definite particle passes through one slit or the other without any interference. Why a particle exhibits wave interference with no WSD, but no interference with a WSD, is the mystery to which Richard Feynmann referred. The particles’ exergies are assumed to be completely dissipated at the detector, so all the transitions have zero utilization (36) and they are all equally probable under Postulate Five. The only difference between the overall transitions with and without the WSD is the probability distributions of their final mixed macrostates, as recorded on the detector screen.

**Figure 10:**
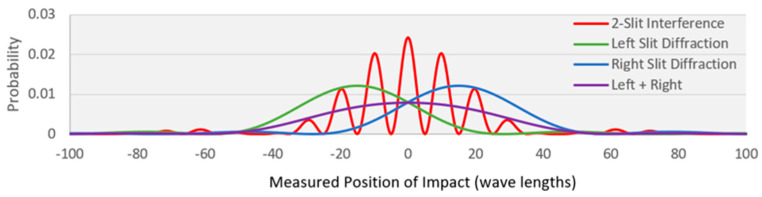
Probability distribution profiles for particle detection from double slits, with and without wave interference, and from single slits.

Figure 10 shows the intensity profiles for the interference and diffraction of light. The profiles are calculated from the equations given in [44] using the parameters shown in Table 2. The intensity profiles also represent the probability distribution profiles for the detection of individual photons. All the profiles are normalized to one and are mapped over a span of 400 possible configuration points based on the model’s detector width and resolution (Table 2).

The red profile in Figure 10 shows the profile for wave interference by the double slits. It describes the probability distribution for individual particle transitions without a WSD. The green and blue profiles show the profile for wave diffraction by a particle from the left or right slit. The purple profile shows the normalized sum of the blue and green profiles. The purple profile describes the probability distribution with the WSD activated, for individual particle transitions from source to detector through one slit or the other.

If the WSD is deactivated, the physical results conform to the red profile’s macrostate model as a wave passing through the slits and producing an interference probability distribution. If the WSD is activated, the results conform to the purple profile’s macrostate model’s two-step transition.

Table 3 shows the calculated configurational entropy changes for each transition based on the probability distribution profiles and Equation (25). Row one shows the entropy for wave interference (red profile) in the absence of the WSD. The entropy change with no WSD is 4.69. The remaining rows show the configurational entropy changes for the two-step transition with the WSD activated.

Row two shows the configurational entropy change for the first transition from the source to a definite particle at one of the two slits. The entropy change is *ln*(2) = 0.69, reflecting the particle’s two equal-probability microstate configurations at the slits. Row three shows the entropy change for the second transition from the definite particle at one of the slits to the detector screen. Its probability distribution is shown in Figure 10 by either the green profile or the blue profile. The entropy for each profile and transition configurational entropy is 5.02. The configurational entropy for the overall two-step transition from the source to the detector screen with the WSD is 5.71 (row 4), equal to the sum of the two steps. This is greater than the configurational entropy for the single-step transition without the WSD (row 1).

With no WSD, the double slits’ symmetry imposes symmetry on the macrostate model. The particles pass through the pair of slits symmetrically with wave-like interference. This is represented by the red profile, with a configurational entropy of 4.69. With the WSD activated, the double slits’ symmetry is broken, and the particle has an opportunity to pass asymmetrically through one slit or the other. The asymmetrical transition’s overall two-step transition is represented by the purple profile, with a configurational entropy of 5.71. With the WSD activated, the system spontaneously breaks its symmetry by selecting one slit or the other to produce the higher-entropy no-interference macrostate, in compliance with MaxEnt (Corollary 4-3).

### 5.2. Configurational Refinement and Replication

In a groundbreaking experiment in 1952, Stanley Miller and Harold Urey demonstrated that energy input to a mixture of simple gases can produce an array of amino acids [45]. More recently, Karo Michaelian and his colleagues have revealed detailed kinetic steps for the synthesis of various complex molecules from simple inorganic precursors by interaction with ultraviolet light [46]. The abiogenic creation of complex molecules from the action of energy on chemical precursors appears to be a common phenomenon, and it is an important step for the origin of life. The kinetics of abiogenic creation of self-replicating nucleotides, which are the key to the origin of life, however, is more problematic. The abiogenic origin of self-replicating nucleotides is an active area of investigation [47], but there has been little success in finding a general principle to explain it.

**Figure 11:**
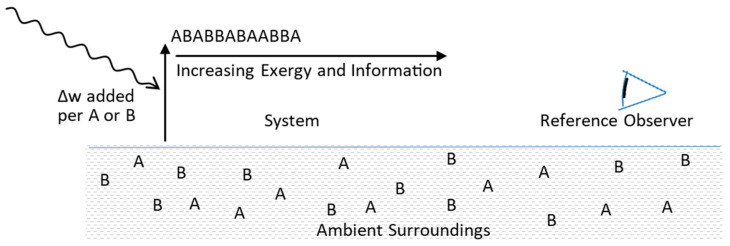
The work of adding an A or B to an array is equal to Δw.

This section provides insight into self-replication based on Corollary 5-4, which provides a spontaneous potential for an external agent to acquire information on a system. To illustrate this, we consider a toy M-type process, shown in Figure 11. The figure illustrates the assembly of a statistical array of A and B monomers by performing the work of taking monomers from the ambient surroundings and adding them to a monomer array. The produced monomer array has a definite, but unknown, configuration of A’s and B’s. We initially assume reversibility, so there is no dissipation, no thermalization, and no random rearrangements of monomers once they are added.

If we assume equal work for adding an A or B to the array, the Boltzmann–Gibbs function describes the addition of A or B at any point as equally probable. If an observer has no additional information, then the macrostate model is the mixed-state array P_ms_ = [½N, ½N … ½N] of length 2^N^, with a configurational entropy and D_KL_ information gap equal to N*ln*(2). The array has a positive exergy, equal to NΔw, but its accessibility is less than this. From (35), the array’s accessibility is given by the following:

(41) $$A=X-C=N\Delta w-k_{B}T_{a}D_{KL}.$$

From Equation (41), reducing the array’s D_KL_ information gap increases its accessibility of energy for work. Corollary 5-4, therefore, provides an external observer with a spontaneous potential to acquire information and reduce the system’s D_KL_ information gap.

One way to reduce the D_KL_ information gap is to create a template that can catalyze the creation of a known sequence. A template is essentially a copy or mirror image of an array to be replicated. Given a template and a procedure that can use it, the template can be used and reused to create an array with a known sequence with zero D_KL_ information gap. From (41), a D_KL_ information gap of zero maximizes the array’s accessible exergy to equal its exergy. Another way to increase the transactional output of accessible exergy is by increasing the array length. Given an appropriate source of energy, Corollary 5-4 provides the spontaneous potential to create a template and procedure to replicate arrays of increasingly greater length, exergy, and information, thereby increasing access to the source’s energy.

The toy model in Figure 11 ignores dissipation and thermalization. In reality, dissipation cannot be eliminated, and thermalization inevitably results in random mutations in the transcription of the template. However, Postulate Five’s Corollaries 5-1 and 5-2 drive the transcription process to increase the transactional output of accessibility by reducing dissipation and random errors from thermalization.

The kinetics for the abiogenic origin of life are exceedingly complex, but Postulate 5 provides a spontaneous potential and a general principle for reproducing increasingly large amounts of accessible exergy and information. It provides the essential selection criterion for guiding the evolution of self-replicating nucleotides to increase their information content and to reduce random transcription errors and mutations.

## 6. Summary and Conclusions

The thermocontextual interpretation (TCI) generalizes Hamiltonian mechanics by adopting the Zeroth and Third Laws of thermodynamics as its first two postulates. The postulates establish a minimum positive temperature of the system’s surroundings, and this defines a system’s ambient temperature. The TCI generalizes mechanics by recognizing ambient temperature, exergy, entropy, and thermal energy as fundamental physical properties of state. The TCI also includes a “no hidden variables” postulate. This postulate establishes a formal distinction between states, which are observable and definite, and transitions between states. These three postulates are TCI’s postulates of state.

The no hidden variables postulate corresponds to Carlo Rovelli’s Completeness Hypothesis 2 of his Relational Quantum Mechanics (RQM) [48]. RQM took an important step by establishing the contextuality of quantum states. TCI’s first two postulates take the next step of providing a unified thermocontextual framework, in which classical mechanics, quantum mechanics, and thermodynamics are all special cases.

Classical, quantum, and thermodynamic states are all ultimately based on observable properties of state. Classical, quantum, and statistical mechanics define states by observations in the absence of thermal noise with respect to hypothetical reference states and observers at absolute zero. Thermodynamics, in contrast, defines states by observations and a reference state in equilibrium with a fixed-temperature thermal bath. They all define states and changes in states with respect to a fixed-temperature reference state—absolute zero for mechanics; a fixed positive temperature for thermodynamics.

Thermodynamics’ positive reference temperature allows it to recognize irreversible change, as expressed by its Second Law of increasing thermodynamic entropy. Nevertheless, thermodynamics, like classical and quantum mechanics, is fundamentally deterministic. Classical thermodynamics, as axiomatically developed by [7] and by [8], strictly deals only with energy states, and it ignores a system’s mechanical details. Thermodynamics, therefore, cannot formally accommodate the spontaneous increase in statistical mechanical entropy associated with reversible mechanical interactions.

The TCI, in contrast, recognizes mechanical mixing and the increase in statistical mechanical entropy as spontaneous processes. The combination of TCI’s three postulates of state, together with the Boltzmann–Gibbs distribution function, establish the fundamental randomness of observations and of transitions generally. States, by definition, are definite and non-statistical, but the transitions between states allow for the random instantiations of new states from multiple microstate potentialities. Random instantiations lead to spontaneous changes associated with wavefunction collapse or random classical transitions.

The TCI formally accommodates random spontaneous transitions by introducing a fixed reference with fixed temperature and information. By convention, the fixed reference temperature is set to the system’s ambient temperature at time zero, and the fixed information is set by perfect ambient measurement at time zero. The fixed reference state is a complete description of the system’s thermocontextual state at time zero. It provides a fixed reference from which spontaneous random transitions due to changes in the ambient surroundings or changes in information can be measured and quantified.

An observer’s actual information defines a system’s macrostate model (24) as an array of resolvable microstate possibilities. The macrostate model is based on the observer’s reference temperature and resolution and on its Bayesian expectation probabilities. Unlike the fixed reference information model, the macrostate model’s information base can increase or decrease in response to measurement and observation or memory losses.

A macrostate model’s accuracy is quantified by a low D_KL_ information gap (28). By convention, the macrostate model is defined as complete at time zero, with a zero D_KL_ information gap. Random changes in a system’s microstate configuration are recorded as increases in the D_KL_ information gap. The TCI also introduces the macrostate model property, accessibility. Whereas exergy is defined by the potential work on the ambient surroundings, which can change during a transition, accessibility is defined and measured by the potential work on the fixed reference state.

TCI’s updated Postulate Four defines the stability of states by minimizing a state’s accessibility. Postulate Four’s Corollary 4-1 is the Second Law of thermodynamics, which describes the irreversible increase in thermodynamic entropy. Corollary 4-2 is closely related to the Second Law. It states that given a fixed ambient temperature, exergy is irreversibly dissipated. Corollary 4-3 is MaxEnt, which is the statistical mechanical interpretation of the Second Law. It describes the spontaneous dispersion and the production of statistical entropy (25) by the random instantiations of new states. Section 5.1 applied MaxEnt to the quantum double-slit experiment to explain why particles spontaneously choose one slit or another and avoid generating an interference pattern when there is a “which-slit” detector in place. The TCI’s Postulate Four fully embraces both MaxEnt and the Second Law as fundamental physical principles, and it provides a unified foundation for mechanics and thermodynamics.

Whereas Postulate Four states that the most stable state has the lowest accessible energy, the updated Postulate Five states that the most stable transition maximizes its output of work and accessible energy. Corollary 5-1 (the growth principle) states that a dissipative system has a spontaneous potential to expand to the environment’s carrying capacity. The maximum efficiency principle (Corollary 5-2) provides a drive for a stationary network of dissipative systems to spontaneously self-organize, and it defines the arrow of functional complexity. Corollaries 5-1 and 5-2 describe two paths by which a dissipative network with a stationary environment can maximize the utilization of its available resources.

Other special cases of Postulate Five address the stability of transitions in response to changing ambient temperature (Corollary 5-3) or associated with an external agent’s gain in information (Corollary 5-4).

Corollary 5-3 (Thermal refinement) describes the spontaneous increase in accessible exergy and the random instantiation of microstate potentialities due to a declining ambient temperature. Cosmic expansion and the symmetry-breaking production of matter over antimatter during the early universe are two outstanding problems of physics. Thermal refinement provides a simple explanation for both cosmic expansion and the predominance of matter over antimatter.

Corollary 5-4 (configurational refinement) is a reformulation of Vopson and Lepadatu’s Second Law of Infodynamics [1]. It describes a transition’s push to minimize the information gap and increase its macrostate model’s accuracy. It is a much stronger statement than the original statement of the Second Law of Infodynamics, which focused on statistical entropy and a description’s precision, without regard to accuracy. Section 5.2 described a simple model to illustrate how Corollary 5-4 can lead to the evolution of self-replicating arrays with increasing exergy, information, and transcription fidelity.

The thermocontextual interpretation represents an alternative to the existing Hamiltonian conceptual framework of classical and quantum mechanics and thermodynamics. By accommodating a positive ambient temperature as a fundamental property of state, the TCI accommodates irreversible dissipation and random instantiation of new states as the fundamental properties of spontaneous processes. It has already shown promise in resolving some of the outstanding questions of physics, chemistry, and biology. Future work is needed to generalize the formalism for quantum mechanics to accommodate non-zero ambient temperatures and random instantiations, and to extend applications of the TCI to other fields, such as economics, social sciences, and machine learning.

## Figures and Tables

**Table 1:** Energy state classifications.

Energy State Classification	Energy Components
Thermal state energy (Q > 0):	E_q_ = (X, Q); E_q_ = X + Q > X
Mechanical state energy (Q = 0):	E_m_ = (X, 0); E_m_ = X
Configurational state energy (Q < 0):	E_c_ = (X, Q); E_c_ = X + Q < X.

**Table 2:** Double-slit interference and diffraction parameters.

Detector Width	Slit Width	Slit Positions	Slit-Detector Separation	Observer’s Resolution
200 λ	7 λ	±15 λ	300 λ	0.5 λ

**Table 3:** Configurational entropies of transitions in double-slit experiment.

	Entropy	Transition (Normalized Probability Distribution)
1	4.69	No WSD—source to detector (red profile)
2	0.69	WSD on—source to one of the slits (50–50%)
3	5.02	WSD on—slit to detector (green or blue profile)
4	5.71	WSD on—overall: source to detector (purple profile)

## Data Availability

No new data were created or analyzed in this study. Data sharing is not applicable to this article.

## Appendix A. State, Macrotate Model, and Transactional Properties

Physical State and State Properties: Based on Perfect Ambient Measurement and Observation		
Ambient Temperature and pressure	T_a_ and P_a_	The minimum temperature and pressure of the system’s physical surroundings with which it could interact.
Ambient Surroundings		Idealized equilibrium surroundings at T_a_ and P_a_
Ambient Reference State	ARS	State in thermodynamic equilibrium with ambient surroundings. It defines zero values for thermocontextual state properties.
Perfect ambient observer		An ambient observer’s resolution is based on ambient temperature. A perfect ambient observer maintains complete information on the thermocontextual microstate.
Total Energy	E_tot_	Total energy relative to reference state at absolute zero (0 K)
Volumetric heat capacity	$C_{v}={\left(\frac{\partial q}{\partial T}\right)}_{v}$	Change in a system’s thermal energy with a change in temperature at a fixed volume.
Ambient-state energy	$E_{\mathrm{as}}=\int_{0}^{T_{a}}C_{v}(T)dT$	Ambient reference state’s energy with respect to zero kelvin.
System Energy	E = E_tot_ − E_as_ = X + Q	System Energy with respect to ambient reference state at T_a_
Thermal energy (heat)	$q=\int_{T_{a}}^{T}C_{v}dT$	System’s thermal energy with respect to ARS. Equals a system’s energy loss as it irreversibly cools to the ambient temperature
Exergy	X ≡ X_m_ + X_q_	System’s reversible work potential on the ambient surroundings. Sum of mechanical and thermal exergy.
Thermal exergy	$X_{q}=\int_{T_{a}}^{T}\frac{T-T_{a}}{T}C_{v}dT$	Reversible work potential by thermal energy on the ARS
Mechanical exergy	X_m_ ≡ X − X_q_	System’s work potential on the ARS after cooling to T_a_
Entropic energy	Q ≡ E − X = q_a_	Thermal energy at T_a_ with zero ambient work potential.
Thermocontextual Entropy	$S\equiv \int_{T_{a}}^{T}\frac{dq}{T}=\frac{Q}{T_{a}}$	dS = dq/T = thermodynamic entropy
Thermal Entropy	σ_q_ = S/k_B_	Dimensionless thermocontextual entropy
Ambient heat	q_a_	Output of heat at ambient temperature
Ambient work	w_a_ = w_out_ + X_out_	Output of work plus exergy to the ambient surroundings
Energy state		Defined by ambient temperature and by reversible measurements of thermally equilibrated system’s temperature, energy, and exergy with respect to the ARS.
Thermocontextual microstate		Mechanical configuration of a system’s irreducibly resolvable parts. Completely described by the perfect ambient observer.
Physical State		Defined by the energy state plus the thermocontextual microstate

Macrostate Model and Transactional ^(1)^ Properties: Based on Fixed Reference Observer and Temperature		
Fixed-Reference Observer		An observer’s resolution is fixed by reference temperature. It creates a macrostate model based on resolvable observations at time zero.
Fixed Reference Temperature	T_ref_	Fixed reference temperature for the measurements of accessible energy and configurational energy. By convention, it is set to ambient temperature at time zero.
Fixed-information reference model		A fixed information model allows tracking random changes in a system. By convention, it is based on perfect ambient observation of a system at time zero.
Macrostate Model	[P_1_, P_2_, … P_Nobs_]	Statistical description of a system’s microstate configuration based on the reference observer’s Bayesian expectation probabilities for the system’s resolvable microstates.
Configurational Entropy	$\sigma_{c}=\sum_{i=1}^{N_{obs}}P_{i}ln(P_{i})$	Describes the macrostate model’s imprecision. The sum is over the microstates resolvable by the reference observer. P_i_ is the reference observer’s Bayesian expectation probability that the system exists in microstate i. Low entropy means high macrostate model precision.
D_KL_ Divergence (information gap)	$D_{KL}(P_{1}|\left|P_{2}\right)=\sum_{i=1}^{N}ln\left(\frac{1}{P_{2,a}}\right)$	Expresses the statistical separation between a reference observer’s macrostate model, with Bayesian probability distribution P_2_, and the system’s actual microstate. The physical state is statistically described by frequentist probability distribution P_1_. The physical state’s actual microstate configuration is ‘a’, with probability P_1,a_=1, and the macrostate model’s Bayesian expectation of microstate ‘a’ is P_2,a_. A high P_2,a_ and low D_KL_ means high accuracy.
Configurational energy	C=C_m_+C_q_=k_B_T_ref_D_KL_ +$\int_{T_{a}}^{T_{ref}}\frac{\left(T-T_{a}\right)}{T}C_{v}dT$	Exergy that is not accessible for work by the reference observer. Mechanical C_m_ is due to incomplete information (D_KL_ > 0) for the microstate. Thermal C_q_ is due to T_ref_ >T _a_.
Accessibility	A ≡ X – C	Energy accessible for work measured at T_ref_. It is based on the reference temperature and the observer’s information.
Reference heat	$q_{ref}\;({\hat{q}}_{ref})$	Output of heat at T_ref_ (per transition)
Reference work	$w_{ref}\;{(\hat{w}}_{ref}$) = w + A_out_	Output of work plus accessibility to T_ref_ (per transition)
Internal work	$w_{int}=\Delta A_{int}$	Work within a network of dissipators of increasing internal accessibility.
Utilization	$\hat{\upsilon }\equiv {\hat{w}}_{ref}+{\hat{w}}_{int}$	Reversible per-transition output of external work at the reference temperature (work plus accessible energy) plus the internal work.
Other transactional properties	$\hat{Q},\;{\hat{\sigma }}_{q},\;{\hat{D}}_{KL},\;\overset{ˇ}{X}$ $,\;\overset{ˇ}{A}$	Per-transition increases in entropic energy, configurational entropy, and D_KL_ information gap; and decreases in exergy and accessibility.
^(1)^ transactional properties are designated by ^ or ˇ to indicate increases or decreases per unit transition.		
